# Tafamidis for autonomic neuropathy in hereditary transthyretin (ATTR) amyloidosis: a review

**DOI:** 10.1007/s10286-019-00625-9

**Published:** 2019-08-12

**Authors:** Márcia Waddington Cruz

**Affiliations:** National Amyloidosis Referral Center-CEPARM, Federal University of Rio de Janeiro, Rio de Janeiro, Brazil

**Keywords:** Tafamidis, Transthyretin, Autonomic dysfunction, Dysautonomia, Orthostatic hypotension, Neuropathy, Amyloidosis

## Abstract

**Purpose:**

Autonomic dysfunction is a very common, early and distressing aspect of hereditary transthyretin (ATTR) amyloidosis leading to significant loss of quality of life and morbidity for patients. Although the clinical variability of ATTR has been well characterized as neuropathic, cardiac or mixed phenotype, the extent of autonomic involvement remains poorly understood. Despite the fact that the autonomic nervous system has not been specifically evaluated in any of the clinical trials of tafamidis, and that, for some primary and secondary endpoints used in these trials, the behavior cannot be separated from non-autonomic items, an attempt was made to use published material to indirectly access the efficacy of tafamidis in treating dysautonomia.

**Methods:**

Literature review summarizing the results of primary and secondary endpoints related to the autonomic features used in the original tafamidis trials, the post hoc publications, and real-world data, on the effect of tafamidis on autonomic dysfunction in patients with ATTR amyloidosis.

**Results:**

There is some evidence that indirectly demonstrates that tafamidis is safe and could slow or arrest the progression of autonomic neuropathy in patients with ATTR amyloidosis, in addition to its well-described effects to ameliorate sensory-motor peripheral neuropathy.

**Conclusion:**

Although the current evidence is scarce, tafamidis might be effective in arresting the progression of autonomic neuropathy in patients with ATTR amyloidosis. Tafamidis might be more effective at the early stage of the disease; however, individual responses must be monitored.

## Introduction

Hereditary transthyretin amyloidosis (ATTR) is a rare disease caused by the deposit of misfolded transthyretin (TTR) monomers as amyloid in several tissues, resulting in a length-dependent axonal degeneration of the peripheral nerves, involving initially and preferentially unmyelinated and small myelinated nerve fibers [6].

Autonomic dysfunction is a very common, early and distressing aspect of TTR amyloidosis affecting the gastrointestinal, urogenital and cardiovascular systems, leading to significant loss of quality of life for the patients, consequentially resulting in a reduction of work capacity and social life. It is also life-risking when considering severe orthostatic hypotension, diarrhea, vomiting and eventually cachexia due to malabsorption as leading causes of death if untreated. Early sexual dysfunction in men is another important psychological aspect to be considered [14].

Although the clinical variability of ATTR has been well characterized as neuropathic, cardiac or mixed phenotype, the extent of autonomic involvement remains poorly understood. Using data from THAOS (a multinational, observational transthyretin amyloid outcomes survey), Barroso and colleagues investigated the prevalence of autonomic symptoms, relationship with different genotypes and phenotypes, and clinical burden for the patients [1]. As of January 2016, a total of 2362 subjects with symptomatic ATTR had been enrolled in THAOS. Of these, 1006 (42.6%) had autonomic dysfunction at enrollment and 1100 (46.6%) at last evaluation (SD time from enrollment to last visit of 2.6 years). The most prevalent symptoms were erectile dysfunction and gastrointestinal and urinary symptoms. Of the 1006 subjects with autonomic failure at enrollment, 355 (35.3%) experienced the related symptom as their first symptom of ATTR. The V30M mutation was associated with a greater prevalence of autonomic dysfunction, shorter time from first ATTR symptom to onset of dysautonomia, and greater clinical burden. Autonomic dysfunction was more prevalent in the neuropathic or mixed phenotypes but can also be found in the classic cardiac phenotype (15.9%). Although very prevalent in the Val30Met early-onset cases, autonomic involvement is usually less prevalent in late-onset Val30Met cases and in many other mutations. The majority of patients in the THAOS database and the ones included in the original trials with tafamidis had the early-onset VaL30Met phenotype.

Tafamidis selectively binds to TTR with negative co-operativity and kinetically stabilizes wild-type native TTR and mutant TTR; tafamidis, therefore, has the potential to halt the amyloidogenic cascade initiated by TTR tetramer dissociation, monomer misfolding, and aggregation [3, 4].

The aim of this manuscript is to discuss relevant autonomic endpoints in relation to the use of tafamidis in clinical trials, and use post hoc analysis as well as in real life to gather experience of different stages of the disease and mutations.

## Methods

We performed a literature review to summarize the impact of tafamidis on autonomic dysfunction in patients with ATTR amyloidosis. Autonomic markers were not specifically evaluated in any of the tafamidis trials. Moreover, the change of primary and secondary endpoints used in these trials, although related to autonomic dysfunction, cannot be separated from other non-autonomic systems. Thus, an attempt was made to include published material to indirectly access the impact of tafamidis in autonomic dysfunction.

Among the primary and secondary outcomes related to autonomic dysfunction used in the tafamidis trials, we focused on nerve function accessed by quantitative sensory test and heart rate variability to deep breathing as measures of small nerve fiber function; The Norfok Quality of Life Questionnaire encompasses several questions related to sphincter control, gastrointestinal and other dysautonomia aspects and their burden, as well as other non-autonomic functions, and, finally, the modified body mass index (mBMI), a well-known prognostic index of disease severity.

## Results

### Tafamidis FX-005 trial

FX-005 was the first tafamidis double-blind, placebo-controlled, multicenter trial. The objective was to evaluate the effect of 18 months of treatment (20 mg daily) on disease progression and its safety. Patients with definite diagnosis of hereditary ATTR, presenting V30M mutations and an autonomic or sensory-motor neuropathy at stage 1 (able to walk without support) of disease, were included. The corresponding primary efficacy endpoint of interest analyzed in the intent-to-treat (ITT; all patients randomized) population was that least-square (LS) mean change from baseline in Norfolk QOL-DN score in the two groups. Another analysis was performed in the population that completed the study (EE). The efficacy evaluable (EE) population was pre-specified assuming a dropout of patients for liver transplantation (LT). A total of 128 patients were randomized, with 69% on the waiting list for LT. From this population, 21% in each treatment arm, active drug, or placebo discontinued to undergo LT. As secondary autonomic endpoints of interest, change from baseline at months 6, 12, and 18 in Norfolk QOL-DN, the summated three nerve tests (Σ3 NTSF), and mBMI were used. The summated three nerve tests small-fiber normal deviate score (Σ3 NTSF nds), a composite of cooling detection threshold, heat/pain detection threshold and heart rate response to deep breathing, ranges from − 11.2 (normal) to 11.2 (total impairment). They can be obtained by quantitative sensory test examination with dedicated equipment [8].

In the ITT population, change from baseline in Norfolk QOL-DN score in the two groups was not significant at month 18. However, in the EE population, significantly less LS mean change from baseline in Norfolk QOL-DN in the tafamidis group was observed (0.1 points vs. 8.9 points, *P* = 0.045). In the analyses of the secondary endpoints in the ITT population at 6, 12, and 18 months, patients receiving placebo demonstrated 5-times greater mean deterioration in small-fiber function [Σ3NTs normal deviates (nds); *P* = 0.05]. In the tafamidis group, BMI significantly increased from baseline at 18 months (LS mean + 39.3) compared to a worsening in patients under placebo (− 33.8). There was an 84% preservation of small-nerve fiber function in the tafamidis-treated group of patients. Adverse events were equally distributed in both treatment groups and were mostly related to the disease itself. The excellent tolerability profile of tafamidis is essential when considering dysautonomia in patients who are very sensitive to gastrointestinal disturbances, cardiovascular and kidney possible adverse events [4].

### Tafamidis FX-006 trial

Fx-006 was an extension study of 12 months, conducted to determine the long-term safety and tolerability of tafamidis and to assess the efficacy of the drug in slowing disease progression. Patients who were randomized to the placebo arm in the previous study were switched to the active drug arm; therefore, two groups of patients were created for comparison: tafamidis–tafamidis and placebo–tafamidis. Efficacy measures were the same as for the previous study and were conducted every 6 months on the ITT population. From the 91 patients who completed the Fx-005 study, 86 (94.5%) were enrolled in Fx-006.

In the tafamidis–tafamidis group, there were no statistically significant differences in the monthly rate of change in measures including Norfolk QOL-DN and Σ3 NTs nds scores comparing the first 18 months of the previous study with the 12 months of the extension study. The monthly rate of change of mBMI dropped in the tafamidis–tafamidis group after entry into Fx-006, but remained higher than at baseline of the previous study. In the placebo–tafamidis group, the rate of progression measured by Σ3 NTs nds score (0.09 for Fx005 and 0.04 for Fx006) was decreased during the extension phase of the study. The deterioration attested by the Norfolk QOL-DN score during Fx-005 was arrested in Fx-006 in this group (monthly rate of change: − 0.16; *P* < 0.01) and there was a positive rate of change of mBMI as opposed to a decline observed in Fx-005 (− 1.77; *P* < 0.0001) [3]. No new safety or tolerability issues were noticed. Taken together, the results indicate that beneficial effects of tafamidis were sustained over a 30-month period and early-start treatment is desirable.

### Tafamidis FX1A-201 trial

In addition to study Fx-005, a parallel study was conducted in patients with amyloidosis due to non-V3OM TTR mutations (excluding V122I) to assess effects of tafamidis on TTR stabilization and clinical outcomes. This was a 12-month study open-label design [11]. As secondary endpoints, Norfolk QOL-DN and mBMI were used. Of the 21 patients enrolled, 18 completed the trial. The baseline demographics of patients demonstrated that this group of non-V30M patients had a longer disease duration (mean 64.7 months) and more advanced age (mean 59.3 months) than the V30M patients enrolled in the previous studies. They represented a considerable heterogeneous population in which eight different TTR non-V3OM mutations were observed with mixed neurological and cardiomyopathy presentation. Mean changes in Norfolk QOL-DN from baseline at months 6 and 12 were negligible. The overall nutritional status was maintained over 12 months with variation between the months 6 and 12 analyses.

### Tafamidis FX1A-303 trial

An open-label extension of these trials is ongoing. The aim is to assess long-term safety and efficacy of tafamidis in ATTRV30M and non-ATTRV30M patients. Ninety-three patients from previous studies were enrolled. Barroso and colleagues performed an interim analysis after up to 6 years of treatment for ATTR-FAP. At the time of the data cut-off, 17 of the 93 were ongoing. The remainder were the ones from countries where tafamidis was still not available by prescription [2]. Norfolk QOL questionnaire and mBMI (modified BMI introducing albumin in the calculation) were evaluated at 6-month intervals during the parent studies and at 12-month intervals during the ongoing extension.

LSMean changes from baseline in TQOL for ATTRV30M patients showed variability, but increased over time. By month 66, LSMean increases from baseline were similar in the tafamidis–tafamidis and placebo–tafamidis groups. By month 48, LSMean increases from baseline for non-ATTRV30M was 24.8. In the same study, a post hoc slope analysis used extrapolated projection of TQOL in patients who were originally under placebo if they would have continued to be under placebo, thus mimicking natural evolution of the disease, and a significant difference was observed, indicating that, although increasing, the change in TQOL compared to no treatment at all is much lower in both groups of patients (treated or not treated previously by tafamidis in FX005). Regarding mBMI at month 66, tafamidis–tafamidis group LSMean change was − 2.7 g/L × Kg/m^2^ (minor numerical decreases). The placebo–tafamidis group of patients showed steady worsening on mBMI relative to baseline during placebo treatment and an improvement (increase) after switching to the active drug, persisting at month 66. (LSMean change at month 18 was − 31.8, and 50.6 at month 66). This long-term analysis expands the favorable safety and efficacy profile of tafamidis in several aspects of autonomic and QOL aspects of ATTR and confirms the growing body of evidence that early initiation of tafamidis is of upmost importance for the clinical outcome [15].

### Post-hoc analysis

In addition to clinical trials, two different post-hoc analysis have been performed to assess the efficacy of tafamidis. Keohane and colleagues described additional post hoc analysis of the data from the pivotal, Fx-005, double-blind trial, employing alternative methods of analyzing change from baseline is NIS-LL (neuropathy impairment score of lower limbs) by itself and in combination with other measures as Σ3 (NIS-LL +  Σ3), a pre-specified efficacy measure of small-nerve fiber function.

The NIS-LL has been described extensively [4]. Briefly, it comprises scores of muscle weakness in the lower limbs, patellar and ankle tendon reflexes, and abnormality of four different modalities of sensation on the big toe, and total score ranges from 0 to 88.

Repeated-measures analysis of change from baseline of the parameters described before was repeated with the addition of baseline as covariate and multiple imputation analysis for missing data by treatment group. It was conducted to account for a numeric difference in baseline NIS-LL between groups (tafamidis, 8.4; placebo, 11.4). Significant treatment group differences in the LS mean change from baseline in NIS-LL +  Σ3 were observed from month 12 to month 18. At baseline the mean NIS-LL + Σ3 in the tafamidis group was 13.9 points and in the placebo group was 17.1 points. The LS mean increase from baseline at month 12 was 1.6 points in the tafamidis patients versus 6.1 points in the placebo-treated ones (treatment group difference of 4.5 points; *P* = 0.0001). The LS mean increase from baseline at month 18 was 3 points in the tafamidis group versus 7.5 points in the placebo group (treatment group difference of 4.5 points; *P* = 0.001). The combination of NIS that is basically a neurological examination, with neurophysiological parameters provided a stronger measure for the heterogeneous aspects of this neuropathy (sensation loss, muscle weakness, decreased reflex, nerve conduction abnormalities and autonomic dysfunction) [8].

We performed a subgroup analysis of the effectiveness of tafamidis in the double-blind trial and its open label extension in a cohort of patients with mild neuropathy at the start of the active treatment (NIS-LL < or = to 10). Mean changes from baseline in NIS-LL and mBMI were 5.3 points and − 7.8 Kg/m^2 ^× g/L at 5.5 years, thus resulting in sustained delay in neurological progression and nutritional status [15].

### Real-world experience

After the approval of tafamidis in Europe in 2011, several centers reported their experience with tafamidis in a real-world context of use. Lozeron and colleagues studied the effect of tafamidis on the disability and safety in late-onset patients with ATTRV30M. Late-onset V30M patients differ from early onset in presentation of disease after 50 years of age, but also in the greater severity of the neuropathy, and as presentation with a cardiomyopathy or mixed phenotype [9]. The study was prospective, non-randomized, carried in a national French reference center for FAP with follow-up at 1 year. They enrolled 37 patients with late-onset V30M FAP patients (mean age was 56.4 years), NIS-LL > 10. The primary study outcome measurements were NIS and disability scores. Among other tests performed, compound autonomic dysfunction test (CADT), including orthostatic hypotension (OH) (normal, 16 to 0) was accessed. Progression in CADT was defined as a decrease > or = 2 [10]. The CADT score deteriorated in 6 of 29 (21%) patients, including 2 with OH during the year follow-up. Two previously normotensive patients developed OH at months 18 and 24 of the follow-up.

Cortese and colleagues reported the monitoring of efficacy and safety of tafamidis in ATTR in Italy. It was a longitudinal multicenter survey in a non-endemic area. Patients from non-endemic areas differ from most of the patients of the pivotal trial in terms of the possibility of mixed (cardiac and neuropathic) presentation, late-onset, later disease stage and non-V30MTTR mutations. Sixty-one patients were followed according to a protocol including general medical, cardiac and neurological assessments at baseline and every 6 months for up to 3 years (not all patients were followed for 3 years). Only 28% had V30M TTR mutation. Mean age of onset was 59 years and 18% had advanced stage at the start of treatment. Patients were assessed by NIS, Kumamoto Scale, FAP stage, Charcot Marie Tooth Scale, PND, different cardiac and echocardiographic parameters and mBMI (albumin introduced in the calculation to compensate for possible edema) [5]. Forty-two (69%) patients had dysautonomia (29, 48% diarrhea; 37, 61% OH; 18, 30% urinary retention; 5, 8% dry eyes and 5 dry mouth). One-third of patients did not show significant progression during 36 months independently of type of mutation and disease stage. Neurological function progressed in the first 6 months but progression was slowed thereafter. A higher mBMI at baseline was associated with better neurological function preservation. Cardiac deterioration was seen in 15% and 30% new onset of cardiac disease. Remarkably, mBMI did not change during 36 months of observation [7]. Thirty-nine patients with evidence of dysautonomia at baseline were assessed by the Kumamoto Scale and followed at 6 months. Autonomic function remained stable in 13 (33%), worsened in 22 (56%) and improved in 4 (10%), including improvement of urinary retention in 2 cases, OH in 1 case, eye and mouth dryness in 2 cases. Of 15 patients without dysautonomia at baseline, 6 developed other symptoms, OH in 4 cases and diarrhea in 4 cases. Two also developed additional symptoms of dry mouth and urinary incontinence. As a total, efficacy of tafamidis on dysautonomia was reached in 26/54 patients (48%) showing normal or stable autonomic function at last follow-up.

Planté-Bordeneuve and colleagues reported the effect of tafamidis in 43 TTR-FAP-treated patients with a variety of mutations (53% non-V30M TTR) at different stages of neuropathy followed long term. Clinical assessments, including BMI and neurological assessment, including neurophysiological variables of small-nerve fiber function, were performed every 6–12 months. The mean duration of follow-up was 2.1 years. Autonomic and small-nerve fiber function measures included in the neurophysiological score of small-fiber (SFNS) were: sympathetic skin response (SSR), heart rate variability with deep breathing (HRDB), laser-evoked potentials (LEP), warm detection threshold during quantitative sensory testing (WDT) and electrochemical skin conductance (ESC) (Sudoscan^®^ [12]) were conducted. The neurological function measured by NIS and mPND deteriorated significantly from 6 to 12 months after treatment. At last follow-up, 29% preserved walking capability without support (PND < 2) and 10% lost walking ability (PND = 4). At 30–36 months, the neurophysiological SFNS worsened significantly, whereas the neurophysiological score for large-nerve fiber function remained stable, indicating the sensitivity of small-nerve fibers to the burden of this disease, especially in this population with a mean baseline NIS-LL of 34.5 (range 2–134.5), reflecting a more severe neuropathy at baseline if compared to the pivotal study. In contrast, a significant increase in BMI at 30–36 months under treatment was observed, indicating a gain in nutritional status [13] (Table 1).

**Table 1:** Endpoints of autonomic significance in tafamidis studies and related cohorts

Study	Autonomic endpoints	Outcome
FX-005 clinical trial	Norfolk QOL-DN score	NS in the ITT population
	mBMI	Positive effect for EE population
	Small-fiber function	Significantly deteriorated in placebo group Significantly preserved in tafamidis group
FX-006 clinical trial	Norfol QOL, mBMI, small-fiber function	Positive rate of change
FX1A-201 clinical trial	Norfolk QOL-DN	Negligible changes
	Nutritional status	Maintained over 12 months
FX1A-303 clinical trial	Norfolk-QOL-DN	The change in TQOL compared to no treatment at all was much lower in both groups of patients
	mBMI	Positive effect for both groups (tafamidis–tafamidis and placebo–tafamidis)
Post-hoc analysis [8]	Combination with NIS-LL with small-fiber unction summated 3	Significantly preserved
Post-hoc analysis [15]	mBMI	Sustained delay in deterioration
Real-world experience [10]	CADT	Deteriorated in 21%
	Development of OH	2 (%) patients
Real-world experience [5]	mBMI	Did not change at 36 months
	Kumamoto Scale	Stable in 33% and improved in 4%
Real-world experience [13]	Neurophysiological score of small-fiber (SFNS)	Deteriorated significantly at 36 months
	BMI	Significant increase in BMI at 30–36 months

## Conclusion

Autonomic abnormalities are frequent among a majority of the clinical presentations of ATTR, and can be the initial presentation of the disease, being very prevalent in patients with the early-onset Val30Met phenotype. Autonomic dysfunction represents a critical part of the burden of the disease, and is generally challenging to treat.

Trials, post hoc analysis, and real-world experience publications presented different results of the efficacy of tafamidis to treat dysautonomia in different groups of patients, from different parts of the world, using many different measures, mostly as indirect secondary endpoints.

There is a consistent beneficial effect of tafamidis to preserve nutritional status, an important prognostic factor in ATTR. The initiation of tafamidis should be considered early in the stage of the disease, when it seems to be more useful. Treatment of ATTR should be individualized, requires experienced practitioners, a multidisciplinary approach and frequent follow-up visits.

Taking into consideration the published data about tafamidis and autonomic dysfunction, there is some evidence indirectly demonstrating that tafamidis is safe and could slow the progression of autonomic neuropathy in some cases, in addition to its well-demonstrated beneficial effects to slow the progression of sensory-motor peripheral neuropathy.
